# Evaluation of a video-based Internet intervention as preparation for inpatient psychosomatic rehabilitation: study protocol for a randomized controlled trial

**DOI:** 10.1186/s13063-016-1417-y

**Published:** 2016-06-13

**Authors:** Jan Becker, Manfred E. Beutel, Katharina Gerzymisch, Dirk Schulz, Martin Siepmann, Rudolf J. Knickenberg, Stefan Schmädeke, Peter Ferdinand, Rüdiger Zwerenz

**Affiliations:** Department of Psychosomatic Medicine and Psychotherapy, University Medical Center of the Johannes Gutenberg-University, Untere Zahlbacher Str. 8, 55131 Mainz, Germany; Media Center of the Johannes Gutenberg-University Mainz, Wallstraße 11, 55122 Mainz, Germany; Psychosomatic Clinic, RHÖN-KLINIKUM Campus Bad Neustadt, Kurhausstraße 31, 97616 Bad Neustadt/Saale, Germany; AHG Clinic for Psychosomatic Rehabilitation Bad Dürkheim, Kurbrunnenstraße 12, 67098 Bad Dürkheim, Germany; Knowledge Media Institute of the University of Koblenz-Landau, Universitätsstraße 1, 56070 Coblenz, Germany

**Keywords:** Inpatient psychosomatic rehabilitation, Online preparation, Randomized controlled trial, Treatment expectation, Outcome expectancy, Internet intervention

## Abstract

**Background:**

Patients’ treatment expectations are a key factor in psychotherapy. Several studies have linked higher expectations to better treatment success. Therefore, we want to evaluate the impact of a targeted video-based intervention on patients’ expectations and the treatment success of inpatient rehabilitation.

**Methods/design:**

All patients who will be referred to inpatient psychosomatic rehabilitation in three clinics will receive a study flyer with information about how to log in to the study platform together with the usual clinic information leaflet. Patients will receive the study information and informed consent upon login and will be randomized into the intervention or the control group. The intervention group (*n* = 394) will get access to our virtual online clinic, containing several videos about inpatient rehabilitation, until their admission to inpatient rehabilitation. The control group (*n* = 394) will receive no special treatment preparation. Questionnaires will be given at study inclusion (T0), two weeks before admission to (T1), and at the end of (T2) inpatient rehabilitation. The primary outcome is the outcome expectancy measured with the Credibility Expectancy Questionnaire at T1. Secondary outcomes include treatment motivation, mental health, work ability, depression, anxiety, and satisfaction with and usage of the Internet platform.

**Discussion:**

We expect the intervention group to benefit from the additional preparation concerning their outcome expectancy. If successful, this approach could be used in the future to enhance the efficacy of inpatient rehabilitation.

**Trial registration:**

ClinicalTrials.gov: NCT02532881. Registered on 25 August 2015.

**Electronic supplementary material:**

The online version of this article (doi:10.1186/s13063-016-1417-y) contains supplementary material, which is available to authorized users.

## Background

Patients’ expectations of treatment are considered an important factor in psychotherapy [1]. According to DeFife and Hilsenroth [2], positive expectations are associated with favorable treatment outcome. In his succinct review, Norcross [3] showed that 15 % of the treatment outcome is determined by expectations. Two main types of expectancy are described in the literature [4]. “Outcome expectancy” refers to a person’s beliefs or feelings about a treatment’s efficacy [5]. “Treatment expectancy” refers to a patient’s expectations about how treatment is delivered, e.g., beliefs about the roles in and the duration of therapy [4]. These two types have been validated in several studies [6]. In line with the findings of Norcross [3], Constantino et al. [5] conducted a meta-analysis with 46 studies. They found a small and positive effect, meaning that higher outcome expectations are associated with a more positive outcome, e.g., symptom reduction. This association was found in other studies as well [7, 8].

Constantino et al. [4], as well as Walitzer and Dermen [9], stated that several techniques, like emphasizing the effectiveness of psychotherapy, very likely enhance a patient’s outcome expectancy of psychotherapeutic treatment. Additionally, Kazdin and Krouse [10] found increased outcome expectations when patients received treatment information including successfully treated case examples, technical terms, and information emphasizing the novelty, scientific evidence, and broad focus of the treatment. Furthermore, Constantino et al. [4] concluded that the delivery of a strong treatment rationale has proved to enhance realistic expectations.

Psychosomatic rehabilitation plays an important role in the German inpatient rehabilitation system, which serves to promote and maintain the ability to work of patients with chronic mental diseases. Psychosomatic rehabilitation is one of the main treatment options in Germany for inpatient psychotherapy; there are approximately 200 clinics. About 60 % of mental disorders have a chronic course, often accompanied by impairments at the workplace and deficits in daily functioning [11]. Patients seeking treatment for their mental health problems, however, may not be motivated for the treatment of work-related impairments [12]. Many patients are not well informed about inpatient rehabilitation, and they are thus unaware that the major goal of rehabilitation is to restore their work capacity [13].

Traditionally, patients have been prepared for inpatient rehabilitation by written material, e.g., brochures. Additional preparatory sessions or discussion groups increased acceptance and knowledge and reduced concerns regarding an upcoming rehabilitation. However, they had no significant effect on treatment outcome [14–16]. Zimmer et al. [17] investigated the effect of a multimodal and partly guided online preparation (VORSTAT) for inpatient psychosomatic treatment, including four different modules (social contacts, information, motivation, and support). Similar to the other studies mentioned, the authors found no difference regarding the improvement of health status after the first two weeks of inpatient treatment between the intervention and the control group. The lack of significant findings, though, may be due to the comprehensive control condition. This assumption is supported by a subsequent study [18]. In a naturalistic observational study, the preparation proved to be effective regarding higher rates of reliable improvement of physical and psychological impairments and social problems in the intervention group compared to a non-randomized control group.

It has been shown that the use of videos as an information source for psycho-oncological patients reduced the fear of cancer treatment and increased satisfaction with the clinic allocation [19, 20]. Several studies have demonstrated that personal cancer stories have a positive influence on the health of recipients [21]. Another study indicated that video information led to reduced anxiety in patients with prostate cancer and increased understanding of their disease and its management [22]. Furthermore, Walthouwer et al. [23] showed that videos are more appreciated, emotionally appealing, and attention-getting than written information.

With the studies of Walitzer and Dermen [9], Kazdin and Krouse [10], and Constantino et al. [4] in mind, we assume that preparing patients for inpatient treatment with a targeted video-based intervention will increase their outcome expectancy and induce more realistic expectations. Furthermore, following up on the promising results of Zimmer et al. [18], we will investigate the influence of our preparation on treatment outcome at the end of inpatient rehabilitation as well. Therefore, this study evaluates a targeted intervention using videos of patients performed by actors and videos of experts to prepare patients for psychosomatic inpatient rehabilitation.

## Methods/design

### Participants

All patients referred to inpatient rehabilitation in one of the three cooperating clinics who are more than 18 years old are eligible for study participation. They will receive the study information together with the usual clinic documents and information about inpatient rehabilitation. Together with the study information, each patient will additionally receive a personal code and a link to the Internet platform (IP). After logging into the IP, patients need to agree to the informed consent presented on the platform. Patients who give their informed consent will be randomized to the intervention or control group and will receive the first questionnaire immediately.

Furthermore, all patients who complete all three questionnaires are eligible to take part in a drawing after completing the third assessment. Patients participating in the drawing can win one of ten cash checks worth 50 € each.

Personal data will be stored at the Study Center of Mental Disorders at the University Medical Center of the Johannes Gutenberg-University Mainz and each cooperating clinic. However, the study assistants with access to the personal data will have no access to the research data collected using questionnaires. Administration of the IP will be managed only by research fellows of the Department of Psychosomatic Medicine and Psychotherapy of the University Medical Center Mainz and the study assistants in the cooperating clinics.

The clinical protocol and informed consent were approved by the Ethics Committee of the Federal State of Rhineland-Palatinate (Germany), which is responsible for the coordinating center in Mainz (Ref. Number 837.192.15 (9960)). Additional approval from Ethics Committees responsible for the cooperating clinics is not necessary if the documents submitted by the coordinating center have been approved by an Ethics Committee. All procedures described in the clinical trial protocol (ClinicalTrials.gov identifier: NCT02532881) follow the International Conference on Harmonization-Good Clinical Practice (ICH-GCP) guidelines and the ethical principles described in the current revision of the Declaration of Helsinki. The trial will be carried out in accordance with local legal and regulatory requirements. A SPIRIT checklist is provided as an additional file (see Additional file 1).

Data privacy and data security of the IP are ensured by several means. First of all, access to the platform is Secure Sockets Layer (SSL)-encrypted. Moreover, the patients do not use their real names to access the platform, which means that their real identity cannot be inferred from the data collected over the platform. Finally, the IP is located on a firewall-protected Web server. As no personal data are stored on the Web server, identification of the user’s identity is not possible.

### Intervention

The targeted intervention was developed in the first part of the trial using qualitative methods [24]. As a first step, we reviewed information provided for rehabilitation patients by the clinics. Patients in rehabilitation clinics and clinical experts from all professions were carefully interviewed using structured and videotaped focus groups and several individual in-depth interviews about the following key issues:

- Information needs and deficits
- Realistic, positive, and negative expectations
- Concerns, fears, and worries regarding psychosomatic rehabilitation

As a second step, in an interdisciplinary work group with the Media Center of the Johannes Gutenberg-University Mainz, findings from content analysis were transformed into patient and expert scripts and video format. These were embedded in a website, with a front page showing the reception area of a virtual clinic with patients and experts standing in the entrance hall. Patients can visit the clinic via the reception area by choosing one of the patients or the expert and watching the videos linked to the respective person. The videos contain important information on various aspects of inpatient rehabilitation. The website was developed by the Knowledge Media Institute of the University of Koblenz-Landau.

The goal regarding concept and design was to present the information in an easy-to-understand and appealing format. Users on the IP are primarily addressed by video instead of plain written text. The image-based approach of video film makes it possible to visualize key parts of the rehabilitation process such as therapy groups as well as psychological states like apprehension about the upcoming treatment in a very immediate and personal manner.

According to this approach, the video contents which will be presented on the IP can be divided into two sets. The first set contains videos about four different fictional patients in a semi-documentary, realistic form. The videos are designed in a brief and personal format to deliver information in an emotional and informative way. Users can browse through the patients’ stories, from the history and decision for rehabilitation to treatment and follow-up. Each video refers to a crucial aspect of the rehabilitation process (fear of confiding in group therapy, being separated from the family, etc.). The roles of these patients are performed by professional actors.

The second set of videos consists of explanatory videos with explicit explanations of concepts, terms, therapies, and other relevant aspects of rehabilitation. This information is presented by fictional experts from different occupation groups working in inpatient rehabilitation. The roles of the experts are performed by actors and staff from the study center on the basis of the scripts previously created by the psychologists of the study center. All topics included in the scripts have been reviewed and revised by corresponding experts in two cooperating clinics and the German Statutory Pension Insurance.

The user can freely choose the videos of both sets. Participants of the intervention group will get access to the IP after they have given their informed consent. Access will expire when subjects are admitted to inpatient rehabilitation.

### Control condition

The control group receives no intervention in addition to the usual inpatient rehabilitation preparation (“as usual”). The preparation contains written information about inpatient psychosomatic rehabilitation and a link to the homepage of the respective clinic.

### Assessment

**Fig. 1:**
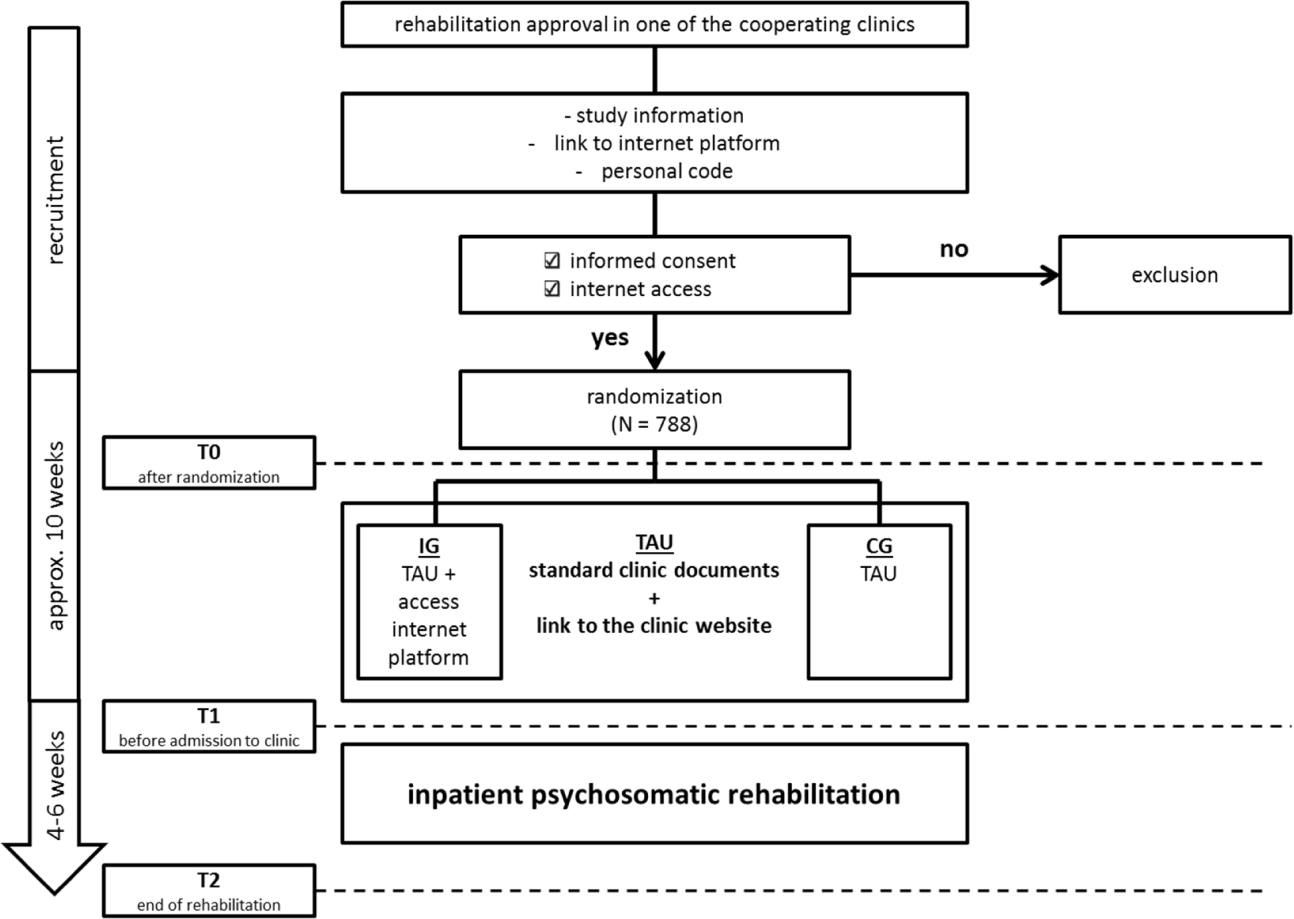
Study design and time points of assessment. *IG* intervention group, *CG* control group, *TAU* treatment as usual

Assessments will be conducted at study inclusion (T0), two weeks before admission to (T1), and at the end of inpatient rehabilitation (T2; see Fig. 1). Questionnaires at T0 and T1 will be given online. At T2 questionnaires can also be administered in paper and pencil form. At T0 patient characteristics like family status and employment will be assessed together with the patient’s history of pre-treatment. Their Internet use will be ascertained as well. The following instruments are used at each time point. The short form of the Patient Health Questionnaire-4 (PHQ-4) [25] will be used to measure depression and anxiety. The mental condition will be assessed at each time point with the Indicators of Rehabilitation Status (IRES-24) [26]. Additionally, patients’ ability to work will be assessed with the Subjective Prognosis of Work Capacity (SPE) [27]. To measure the patient’s expectancies, the Credibility Expectancy Questionnaire (CEQ) [28] will be given at T0 and T1. Furthermore, the Work-Related Therapy Motivation Questionnaire (FBTM) [29], the Questionnaire for Assessment of Rehabilitation Expectancy and Motivation (FREM-17 [30]), and the Patient Questionnaire for Assessment of Rehabilitation Motivation-20 (PAREMO-20 [31]) will be used to assess work-related therapy motivation and treatment motivation at T0 and T1 as well. The use of the IP and websites with clinic reviews and ratings as well as the clinic websites will be measured only at T1. Patients will be asked about their satisfaction with inpatient treatment at T2. All questionnaires and the time points at which they are administered are displayed in Table 1.

**Table 1:** Schematic overview of frequency and scope of the study visits

Visits/scope	First login on platform	After randomization	Before inpatient rehabilitation	End of rehabilitation
Study visits		T0	T1	T2
Written informed consent	X			
Internet use		X		
Pre-treatment		X		
CEQ		X	X	
FBTM		X	X	
FREM-17		X	X	
PAREMO-20		X	X	
PHQ-4		X	X	X
IRES-24		X	X	X
SPE		X	X	X
IP usage			X	
Use of websites with clinic reviews and clinic website			X	
BADO			X	X
Patient Satisfaction Questionnaire-8 (ZUF-8)				X

*BADO* Basis documentation

### Objectives and hypotheses

The primary objective of our trial is to determine the effect of our IP on participants’ outcome expectancy concerning inpatient psychosomatic rehabilitation. We hypothesize a more positive outcome expectancy measured with the CEQ in the intervention group than in the control group at T1. We further hypothesize that the intervention group will have a greater work-related therapy motivation measured with the FTBM than the control group at T1.

### Outcomes

The primary outcome in the study is the outcome expectancy (CEQ) measured at T1 (two weeks before admission to inpatient rehabilitation).

Key secondary outcomes are:

1. Work-related therapy motivation (FBTM) at T1
2. Treatment motivation (FREM-17 and PAREMO) at T1
3. Treatment credibility (CEQ) at T1
4. Satisfaction with and usage of IP at T1
5. Mental condition (IRES-24) at T2 (end of rehabilitation)
6. Satisfaction with inpatient rehabilitation (ZUF-8) at T2
7. Subjective prognosis of work ability (SPE) at T2
8. Depression and anxiety (PHQ-4) at T2
9. Functioning in everyday life (IRES-24) at T2
10. Perceived advantage of aftercare at T1 and T2

Predictors of outcome are:

1. Therapy motivation (FREM-17 and PAREMO-20) at T0 (after randomization)
2. Internet use and technology affinity at T0
3. Use of websites with clinic reviews and ratings as well as use of the cooperating clinics’ websites at T1
4. Previous, especially rehabilitation, treatments prior to the current inpatient rehabilitation at T0

We have defined four predictors of outcome which may have an additional impact on outcome, potentially confounding the impact of patients’ expectations. As it has a major influence on the therapy success, treatment motivation will be controlled [32]. We further think that strong Internet use and technology affinity may influence the use of our platform [33]. We will control for the use of websites with clinic reviews and ratings as well as the use of the cooperating clinics’ websites. Finally, we need to know if patients have had prior inpatient (rehabilitative) treatments, as previous experience may influence expectations. All predictors will be used as control variables in additional explorative analyses after testing our primary and secondary outcomes.

### Sample size calculation

Effect sizes will be calculated to describe treatment effects. We anticipate small effects (*d* = .24) concerning the primary outcome [4]. Based on a level of significance of *p* < .05, a statistical power of .80, one covariate, and two groups, *N* = 788 patients (394 in the control group and 394 in the intervention group) are necessary to detect a between-group effect (a priori power analysis of an ANCOVA with GPower version 3.1). With an expected participation rate of 32 % [34], we need to ask *N* = 2459 patients for study participation to reach this sample size. Considering that there were 4496 patients in the three cooperating clinics per year, we need to recruit patients for at least seven months to reach the planned sample size.

### Randomization

Participants will be assigned to the intervention and control groups using a stratified block randomization with a ratio of 1:1. Patients will be stratified based on the clinic they will be in during their inpatient rehabilitation. Randomization will be conducted with an algorithm implemented on the IP.

### Statistical methods

Analysis of covariance will be used to evaluate the CEQ scores at T1 between the intervention and control groups with CEQ score at T0 as the covariate. The first eight secondary outcomes will be analyzed with analyses of covariance in analogy to the primary outcome. The last two secondary outcomes will be reported with descriptive statistics. Intention-to-treat analyses with multiple imputations to replace missing data as well as per-protocol analyses will be conducted.

## Discussion

Patients’ expectations are known key factors in psychotherapy. Several studies have confirmed the influence of patients’ expectations on the therapy outcome. Moreover, it is known that expectations can be specifically influenced, e.g., by providing a good and convincing treatment rationale [35]. Thus, not only expectations as a key factor but also the therapy outcome itself can be influenced by preparation. Although this could offer an easy way to enhance the results of psychotherapy, the few intervention studies in Germany which have investigated the influence of preparation on patients’ expectations and therapy outcome showed mixed results [14, 17, 18].

In line with previous findings [20, 35], we expect a targeted intervention based on video clips embedded in a virtual online clinic format to enhance positive and realistic patients’ outcome expectations and thereby improve treatment participation and outcome. Studying the connection between an emotional approach and a greater effectiveness in recognition, Cahill et al. [36] showed that emotional video clips were remembered more often than neutral videos. Therefore, we deliberately chose a video-based approach with actors and scripted patient stories conveying common disorders and targeted information about the treatment process, measures, roles of patients and therapists, and treatment outcomes in an emotional and personal way.

We wanted to design our intervention in a modern and trendsetting way to enhance the chances for continuation and dissemination if it proves to be effective. To verify our assumption that videos are an appropriate and well-accepted medium to convey information, we will collect objective data in addition to the data assessed by questionnaires. Therefore, patients’ user behavior will be tracked on the platform and linked with the treatment results. Tracking includes the time spent on the website, the number and types of videos, and the duration of all videos watched by each patient.

Overall, our intervention will use a novel and highly advanced approach to prepare rehabilitants for their inpatient treatment. We intend to determine if such a modern approach can improve patients’ expectations and the treatment outcome. If our intervention proves to be effective, it could be used as a blueprint for a new preparation method for psychosomatic rehabilitants in Germany. It could be used along with the usual written information every patient receives before his admission to inpatient rehabilitation. It would easily be possible, though, to adjust the design to fully substitute the written information. It is also imaginable that the intervention can be adopted for other treatments, like psycho-oncology or orthopedic or cardiovascular diseases. In summary, this trial will provide further insight into the process of preparing patients properly for psychosomatic rehabilitation, which is the most common inpatient psychotherapeutic treatment option in Germany. This is an important approach, particularly against the background of an increasing number of people diagnosed with mental disorders in Germany.

### Trial status

The first patients will be enrolled to the study in September 2015. Assessments for the last included patients are expected to be completed by August 2016.

## Abbreviations

CEQ, Credibility Expectancy Questionnaire; FBTM, Work-Related Therapy Motivation Questionnaire; FREM-17, Questionnaire for Assessment of Rehabilitation Expectancy and Motivation; IP, Internet platform; IRES-24, Indicators of Rehabilitation Status-24; PAREMO-20, Patient Questionnaire for Assessment of Rehabilitation Motivation-20; PHQ-4, Patient Health Questionnaire-4; SPE, Subjective Prognosis of Work Capacity; ZUF-8, Patient Satisfaction Questionnaire-8; BADO, Basis documentation

## Additional file

Additional file 1: SPIRIT checklist. Overview of important items of a clinical trial and their placement in the manuscript. (DOC 122 kb)
